# Implementation Method and Bench Testing of Fractional-Order Biquadratic Transfer Function-Based Mechatronic ISD Suspension

**DOI:** 10.3390/s25144255

**Published:** 2025-07-08

**Authors:** Yujie Shen, Dongdong Qiu, Haolun Xu, Yanling Liu, Kecheng Sun, Xiaofeng Yang, Yan Guo

**Affiliations:** 1 Automotive Engineering Research Institute, Jiangsu University, Zhenjiang 212013, China; shenyujie@ujs.edu.cn (Y.S.); 2222338015@stmail.ujs.edu.cn (D.Q.); sunkc@ujs.edu.cn (K.S.); 2 BYD Auto Industry Co., Ltd., Shenzhen 518118, China; stevenxu99@hotmail.com; 3 School of Automotive and Traffic Engineering, Jiangsu University, Zhenjiang 212013, China; yangxf18@ujs.edu.cn; 4 School of Automotive Engineering, Changzhou Institute of Technology, Changzhou 213032, China; klausguo0526@126.com

**Keywords:** vehicle suspension, mechatronic inerter, fractional-order biquadratic transfer function, implementation method

## Abstract

To address the challenge of physically realizing fractional-order electrical networks, this study proposes an implementation method for a mechatronic inerter–spring–damper (ISD) suspension based on a fractional-order biquadratic transfer function. Building upon a previously established model of a mechatronic ISD suspension, the influence of parameter perturbations on the suspension’s dynamic performance characteristics was systematically investigated. Positive real synthesis was employed to determine the optimal five-element passive network structure for the fractional-order biquadratic electrical network. Subsequently, the Oustaloup filter approximation algorithm was utilized to realize the integer-order equivalents of the fractional-order electrical components, and the approximation effectiveness was analyzed through frequency-domain and time-domain simulations. Bench testing validated the effectiveness of the proposed method: under random road excitation at 20 m/s, the root mean square (RMS) values of the vehicle body acceleration, suspension working space, and dynamic tire load were reduced by 7.86%, 17.45%, and 2.26%, respectively, in comparison with those of the traditional passive suspension. This research provides both theoretical foundations and practical engineering solutions for implementing fractional-order transfer functions in vehicle suspensions, establishing a novel technical pathway for comprehensively enhancing suspension performance.

## 1. Introduction

With the continuous advancement of new energy vehicles and the increasing popularization of autonomous driving technologies, the requirements for the ride comfort of passenger vehicles have been constantly elevated [1,2]. As a fundamental component of the vehicle chassis system, the suspension system performs critical functions such as providing body support and road shock absorption, which directly affect the ride comfort, handling stability, and driving safety [3,4,5,6]. Although traditional passive suspensions remain dominant in the market owing to their structural simplicity, high reliability, and cost-effectiveness, their fixed stiffness and damping parameters restrict performance optimization under varying road conditions and driving requirements [7]. In contrast, semi-active and active suspensions can dynamically adjust the damping characteristics or apply active forces to improve the performance but face challenges including high energy consumption, complex control algorithms, and elevated manufacturing costs, restricting their widespread adoption [8,9,10].

In 2002, Professor Smith put forward the concept of an “inerter” [11], offering a brand-new idea for suspension design. As a two-terminal mechanical element, an inerter generates an output force proportional to the relative acceleration between its terminals, exhibiting functional equivalence to a capacitor in electrical systems [12]. This innovative concept not only refined the mechanical–electrical analogy theory but, more significantly, also introduced established electrical network synthesis methods into mechanical system optimization. Building upon this theoretical foundation, subsequent research has developed various inerter implementations including rack-and-pinion [13], fluid-based [14], living-hinge [15], and ball-screw [16] types, substantially expanding the suspension design possibilities. Liu [17] experimentally demonstrated, using three distinct topological configurations, that inerter-based heavy vehicle suspensions significantly reduce road damage. Further investigations by Li [18] revealed that such suspensions maintain exceptional vibration isolation performance across varying road conditions and speeds while exhibiting characteristic optimal speed effects. These findings collectively substantiate the positive impact of inerters on enhancing the overall suspension performance.

The introduction of inerter elements has led to an increase in the number of suspension components, prompting a shift in the design methodology for inerter-based suspensions from structural approaches to systematic impedance-based design frameworks [19]. Within the theoretical framework of passive network synthesis, the complexity of realizing biquadratic and bicubic transfer functions is closely tied to the number of required components. Classical theory indicates that implementing a biquadratic transfer function typically requires up to eight passive components, while a bicubic transfer function may necessitate as many as twelve. In recent years, scholars have achieved a series of groundbreaking advancements in minimal-component realization: For biquadratic transfer functions, Jiang [20,21] first reduced the required number of components to six and subsequently optimized this to five. Wang [22] further pushed the theoretical limits by systematically establishing the necessary and sufficient conditions for implementations using four and even three components. Research by Hu [23] demonstrated that while higher-order, integer-order transfer functions can markedly enhance the vibration isolation performance of suspension systems, the associated complexity and implementation difficulties in network synthesis increase dramatically. Regarding higher-order bicubic transfer functions, Zhang [24] successfully developed a seven-component synthesis scheme, a record later surpassed by researchers who achieved implementations with only six [25] and five components [26]. It is particularly noteworthy that as the number of components decreases, the mathematical constraints that the transfer function must satisfy grow exponentially, posing significant challenges for practical engineering applications. This inherent contradiction severely restricts the practical implementation of high-performance ISD suspension systems in engineering contexts.

Fractional calculus theory offers innovative solutions to these challenges [27,28,29]. Comparative studies have demonstrated that fractional-order models more accurately characterize the dynamic behaviors of physical components, including their capacitive and inductive properties [30,31,32]. Their mechanical applications include modeling power-law phenomena in fluid inerters [33] and the viscoelastic characteristics of magnetorheological dampers [34]. In the field of electrical circuit systems, Lin [35] investigated fractional-order RC circuit models employing Caputo and Caputo–Fabrizio derivatives, demonstrating that fractional-order modeling approaches can more accurately characterize real-world RC circuit behaviors. Jin [36] proposed an uncertain fractional-order multi-objective optimization framework based on reliability criteria, applying it to RC circuit models and deriving both analytical solutions and numerical optimization results. Hua [37] examined a vibration mitigation system incorporating a three-element electrical network structure composed of fractional-order inductors, fractional-order capacitors, and resistors, subsequently analyzing the effects of parametric variations on the system’s damping performance. However, the existing studies on fractional-order vehicle suspensions predominantly focus on control theories or dynamic modeling, with the insufficient exploration of the use of fractional-order transfer functions for external circuit design in mechatronic inerter-based suspensions. Building upon our prior research on vibration suppression using a fractional-order biquadratic electrical network to produce vehicle mechatronic ISD suspensions [19], this study explored an implementation method for such suspensions.

This paper is structured as follows: Section 2 presents the system model of the fractional-order biquadratic ISD suspension along with its key parameters. Section 3 analyzes the impact of component parameter variations on the dynamic performance characteristics of the suspension. Section 4 describes how we realized the fractional-order electrical components using the Oustaloup filter algorithm and performed simulation validation. Section 5 describes how we constructed a prototype of the mechatronic ISD suspension based on the fractional-order biquadratic transfer function for experimental verification.

## 2. The Fractional-Order Biquadratic Transfer Function-Based Mechatronic ISD Suspension

**Figure 1:**
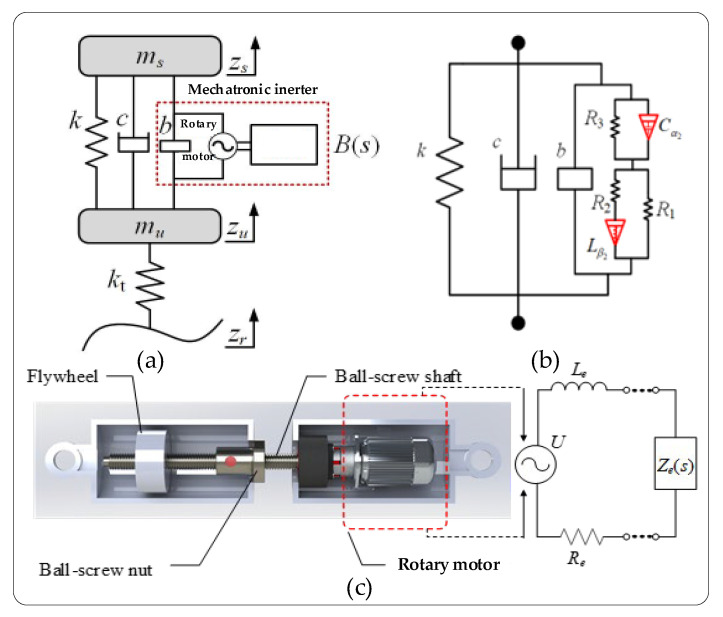
(**a**) Quarter-vehicle model; (**b**) Optimal realization of passive structure; (**c**) Mechatronic inerter device.

Based on our prior research [19], optimization algorithms [38,39,40,41] can be employed for the parametric optimization of a vehicle mechatronic ISD suspension system based on fractional-order biquadratic transfer functions. Figure 1 shows the schematic of the fractional-order biquadratic transfer function-based mechatronic ISD suspension system:

The admittance expression *Y*(*s*) of the external electrical network connected to the motor terminals is formulated as follows:

(1) $$Y_{e}\left(s\right)=\frac{As^{\alpha +\beta }+Bs^{\alpha }+Cs^{\beta }+D}{Es^{\alpha +\beta }+Fs^{\alpha }+Gs^{\beta }+H}$$

In Equation (1), coefficients *A*, *B*, *C*, *D*, *E*, *F*, *G*, and *H* represent the parameters of the fractional-order biquadratic function, while *α* and *β* denote the orders of the Laplace operator.

Based on the kinematic analysis of the sprung and unsprung masses, a quarter-vehicle suspension vibration model was established as shown in Figure 1b, with its corresponding Laplace-domain equations expressed as follows:

(2) $$\left\{\begin{array}{c} m_{s}s^{2}Z_{s}+\left[k+cs+sB(s)\right]\left(Z_{s}-Z_{u}\right)=0\\ m_{s}s^{2}Z_{u}-\left[k+cs+sB(s)\right]\left(Z_{s}-Z_{u}\right)+k_{t}\left(Z_{u}-Z_{r}\right)=0 \end{array}\right.$$

*m_s_* and *m_u_* denote the vehicle’s sprung and unsprung masses, respectively; *z_s_* and *z_u_* represent their respective vertical displacements; *k* represents the stiffness of the supporting spring in the suspension system; *c* represents the viscous damping coefficient; *b* represents the inertance value of the mechanical inerter; *B*(*s*) represents the velocity-domain impedance of the mechatronic inerter; *k_t_* represents the tire’s equivalent stiffness; *z_r_* represents the road excitation input; and the Laplace transforms of *Z_s_*, *Z_u_*, and *Z_r_* are represented by *z_s_*, *z_u_*, and *z_r_*, respectively.

The parameters of the vehicle mechatronic ISD suspension are presented in Table 1.

## 3. Parameters’ Effects on Suspension’s Dynamic Performance

### 3.1. Influence of Spring and Damper Parameters on Suspension’s Dynamic Performance

**Figure 2:**
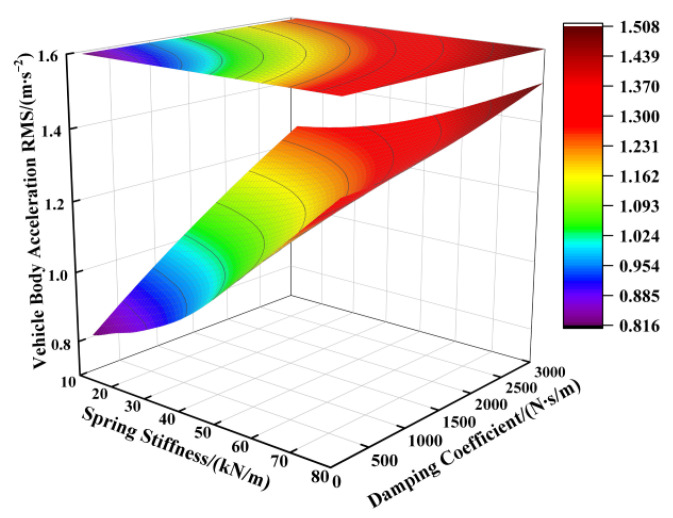
Influence of spring stiffness and damping coefficient on vehicle body acceleration.

**Figure 3:**
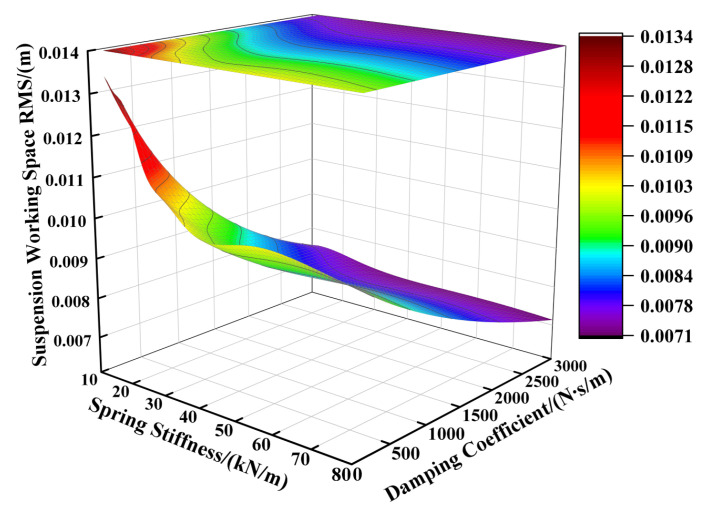
Influence of spring stiffness and damping coefficient on suspension working space.

**Figure 4:**
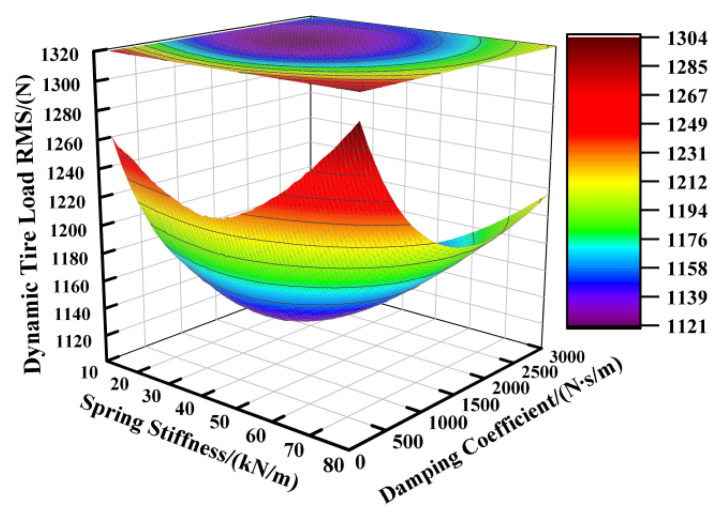
Influence of spring stiffness and damping coefficient on dynamic tire load.

Based on the suspension component parameters specified in Table 1, the supporting spring’s stiffness was varied from 12,000 N/m to 80,000 N/m and the damping coefficient from 100 N∙s/m to 3000 N∙s/m, while keeping other parameters constant. Figure 2, Figure 3 and Figure 4 illustrate the influence of these spring stiffness and damping coefficient variations on the vertical performance metrics of the fractional-order ISD suspension.

Figure 2 illustrates the impact of the spring stiffness on the vehicle body acceleration of the fractional-order ISD suspension. The results indicate a positive correlation between the spring stiffness and vehicle body acceleration, where increasing the stiffness significantly elevated the acceleration levels, while reducing the stiffness yielded lower acceleration values. The damping coefficient showed limited effectiveness in controlling the vehicle body acceleration under high-spring-stiffness conditions, with its influence becoming apparent only when the spring stiffness was sufficiently reduced. Consequently, examining Figure 2 leads us to the conclusion that minimizing both the spring stiffness and damping coefficient reduced the vehicle body acceleration. However, the operational laws shown in Figure 3 reveal another critical issue: the reduction in the spring stiffness and damping coefficient will aggravate the deterioration of the suspension working space, thereby increasing the probability of the suspension reaching the limit. From Figure 4 we can further identify an optimal parameter range, demonstrating that the suspension achieved reduced dynamic tire loads when the supporting spring stiffness was maintained between 15,000 N/m and 55,000 N/m, coupled with damping coefficients ranging from 1500 N·s/m to 2500 N·s/m.

### 3.2. Influence of Inertance on Suspension’s Dynamic Performance

**Figure 5:**
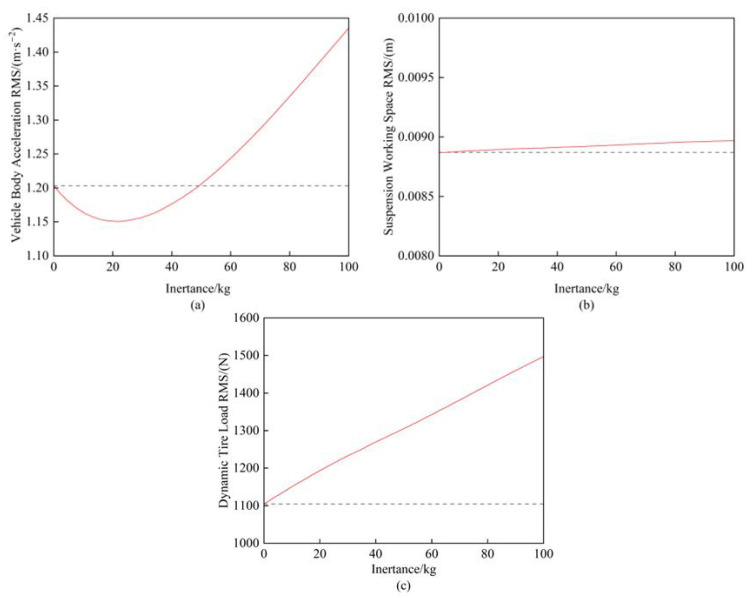
(**a**) Influence of inertance on vehicle body acceleration; (**b**) Influence of inertance on suspension working space; (**c**) Influence of inertance on dynamic tire load.

Based on the parameters listed in Table 1, the inertance was varied from 0 kg to 100 kg while keeping the other parameters constant. Figure 5 illustrates the variation patterns of the vertical suspension performance metrics with changing inertance values, where the black dashed line represents the performance indicators when the inertance equaled 0 kg.

First, Figure 5b demonstrates that the inertance value had a negligible influence on the suspension working space. Meanwhile, Figure 5a reveals that an inertance ranging from 0 kg to 50 kg provided beneficial effects on the vehicle body acceleration, with the minimum acceleration achieved at 20 kg. However, Figure 5c shows a strictly linear increase in the dynamic tire load with higher inertance values, indicating that the smallest feasible inertance should be selected when the equivalent vehicle body acceleration performance is maintained.

### 3.3. Influence of Electrical Resistances on Suspension’s Dynamic Performance

**Figure 6:**
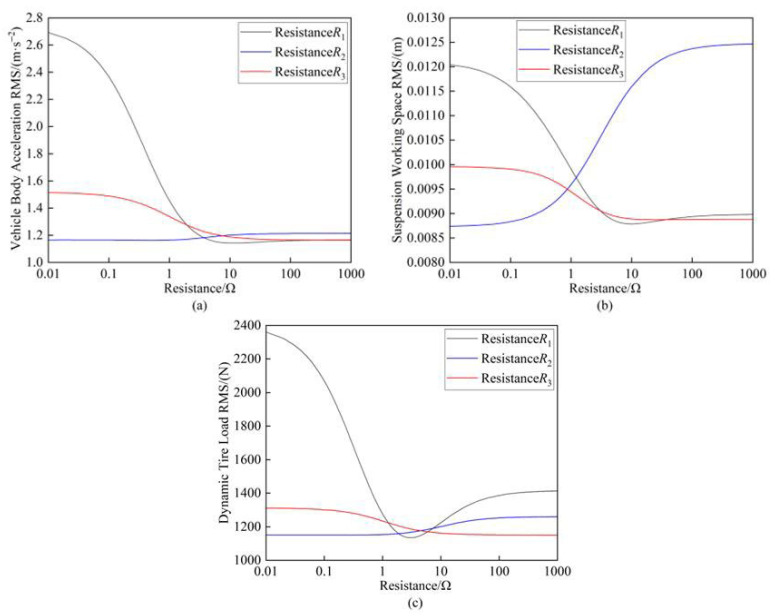
(**a**) Influence of resistance on vehicle body acceleration; (**b**) Influence of resistance on suspension working space; (**c**) Influence of resistance on dynamic tire load.

Based on the electrical component parameters specified in Table 2, the resistance values of *R*_1_, *R*_2_, and *R*_3_ were individually varied from 0.01 Ω to 1000 Ω while keeping other parameters constant. Figure 6 presents the variation patterns of the vertical suspension performance metrics under the independent modifications of these three resistances.

The resistance range of 0.01 Ω to 1000 Ω was further subdivided into three intervals: [0.01 Ω–1 Ω], [1 Ω–10 Ω], and [10 Ω–1000 Ω]. As shown in Figure 6, all three suspension performance metrics demonstrated high sensitivity to variations in resistance *R*_1_. Notably, when *R*_1_ fell within the [1 Ω–10 Ω] range, the performance metrics exhibited rapid deterioration. Beyond this interval (10 Ω–1000 Ω), both the vehicle body acceleration and suspension working space gradually stabilized, while the dynamic tire load showed further degradation. Comparative analysis revealed that resistance *R*_2_ yielded the most favorable performance outcomes within the [0.01 Ω–1 Ω] range when compared to *R*_1_ and *R*_3_. However, increasing *R*_2_ to higher values [10 Ω–1000 Ω] significantly exacerbated the suspension working space deterioration. Among the three resistances, *R*_3_ had the least pronounced effect on the suspension performance. Its influence followed a consistent decreasing trend across all the metrics as the resistance increased from small values [0.01 Ω–1 Ω] to large values [10 Ω–1000 Ω].

### 3.4. Influence of Fractional-Order Capacitance on Suspension’s Dynamic Performance

**Figure 7:**
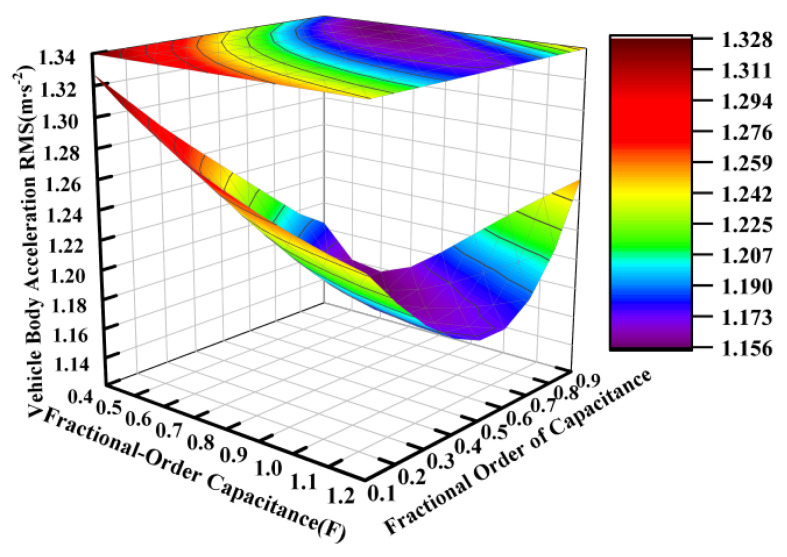
Influence of fractional-order capacitance on vehicle body acceleration.

**Figure 8:**
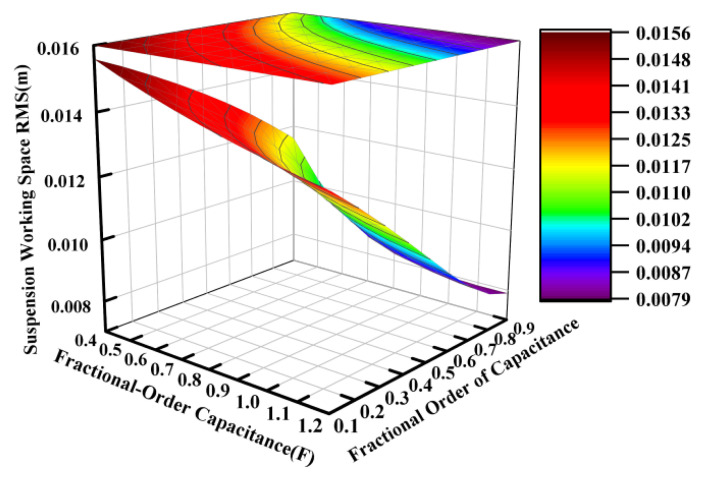
Influence of fractional-order capacitance on suspension working space.

**Figure 9:**
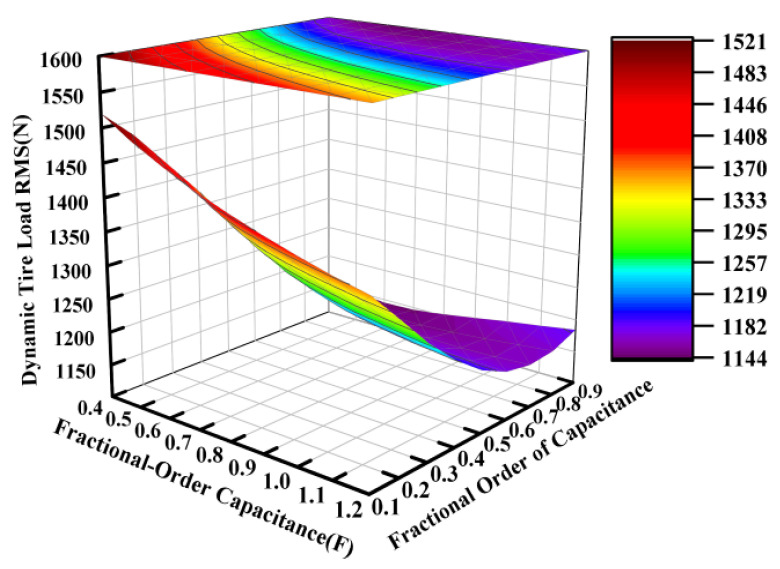
Influence of fractional-order capacitance on dynamic tire load.

Based on the electrical component parameters specified in Table 2, the capacitance value was varied from 0.04 F to 0.12 F while the fractional order was adjusted between 0.1 and 0.9, with the other parameters held constant. Figure 7, Figure 8 and Figure 9 present the variation patterns of the vertical suspension performance metrics under changes in both the fractional-order capacitance value and its corresponding fractional order for the ISD suspension.

Figure 7, Figure 8 and Figure 9 demonstrate that the fractional order of the capacitance significantly influenced the suspension dynamics. At a fixed capacitance value, the optimal selection of the fractional order, particularly within the range of 0.6 to 0.9, could effectively enhance the vibration isolation performance. While the dynamic tire load exhibited a relative insensitivity to capacitance value variations within the tested range of 0.04 to 0.12 farads, intermediate values between approximately 0.06 and 0.10 farads are recommended to simultaneously optimize both the vehicle body acceleration and suspension working space.

### 3.5. Influence of Fractional-Order Inductance on Suspension’s Dynamic Performance

**Figure 10:**
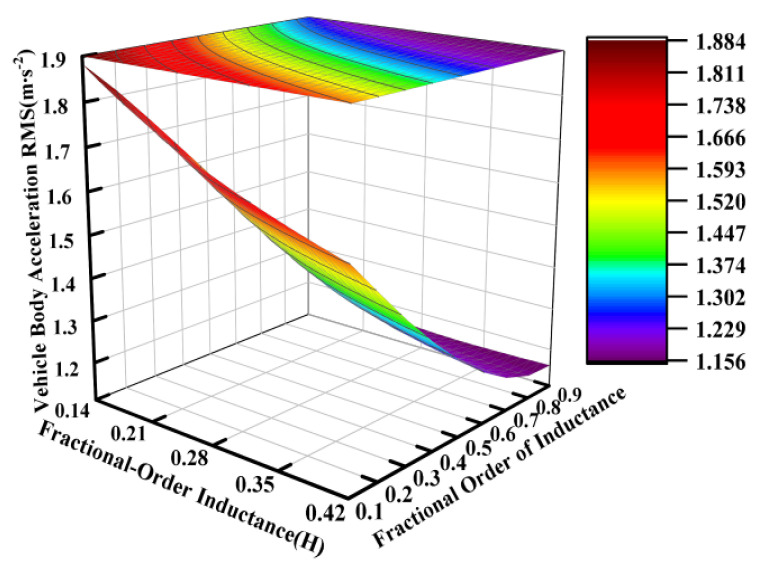
Influence of fractional-order inductance on vehicle body acceleration.

**Figure 11:**
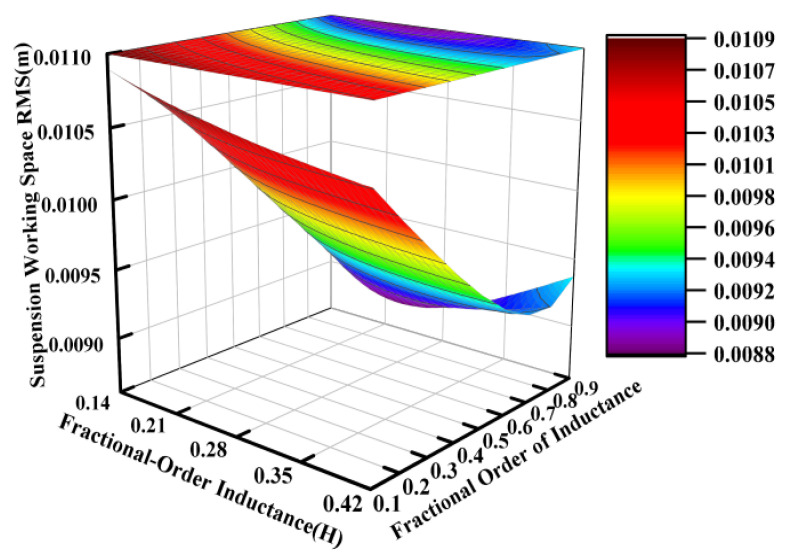
Influence of fractional-order inductance on suspension working space.

**Figure 12:**
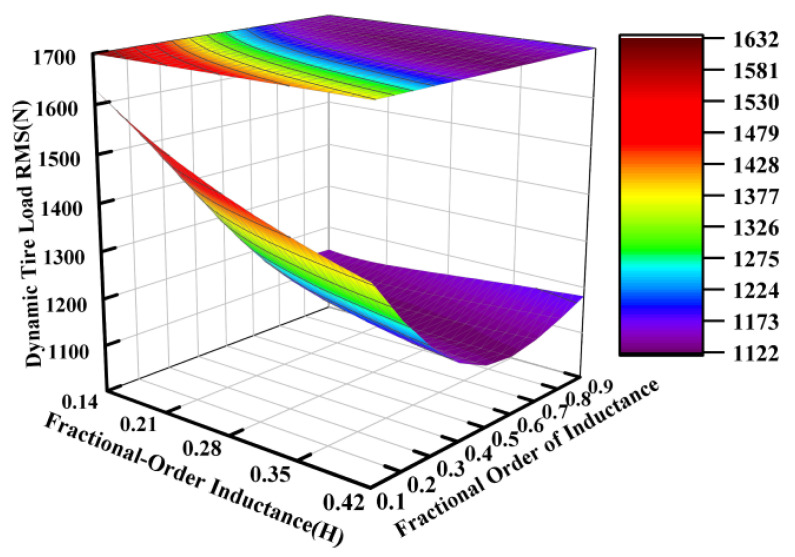
Influence of fractional-order inductance on dynamic tire load.

Using the electrical parameters from Table 1 as a baseline, parametric studies were conducted with inductance values ranging from 0.14 H to 0.42 H and fractional orders varying between 0.1 and 0.9, while keeping other parameters constant. Figure 10, Figure 11 and Figure 12 systematically present the effects of fractional-order inductance variations on the vertical dynamic performance metrics of the ISD suspension.

Similarly to the operational principles observed for the fractional-order capacitance and its order, the fractional order of the inductance also carried significant weight in influencing the vibration isolation performance of the suspension system. This effect is particularly pronounced in Figure 11.

In conclusion, the parameter influence analysis of the fractional-order biquadratic ISD suspension demonstrated that selecting a spring stiffness of *k* = 53,100 N/m and damping coefficient of *c* = 1000 N·s/m effectively balanced the impacts of the vehicle body acceleration and suspension working space on the overall suspension performance. The results of the parameter perturbation analysis described in this section are consistent with those of Pareto optimization in our research [19], achieving closed-loop verification, which enhances the robustness of the fractional-order model. Furthermore, the weights of the influence of the fractional orders (*α* = 0.82, *β* = 0.88) on the suspension performance were validated. These findings provided guidance for the subsequent experimental phase. The highly sensitive parameter *R*_1_ required the use of high-precision components, as minor changes in the parameter had a significant impact on the overall performance of the suspension.

## 4. The Implementation of the Fractional-Order Biquadratic Transfer Function-Based Mechatronic ISD Suspension

### 4.1. Realization Methods for Fractional-Order Electrical Components

In Section 2, we present the structure diagram of the external electrical network for the ISD suspension based on the fractional-order biquadratic transfer function. It should be noted that practical engineering implementations typically require approximating fractional-order systems with finite-dimensional integer-order systems. This study focused on integer-order continuous model approximation methods for fractional-order systems, specifically involving the design of integer-order continuous filters that optimally approximate the dynamic characteristics of the original fractional-order model when excited by unknown input signals.

Fractional-order control systems can be characterized by transfer functions of the following form:

(3) $$G(s)=\frac{b_{m}s^{\beta_{m}}+b_{m-1}s^{\beta_{m-1}}+\ldots +b_{0}s^{\beta_{0}}}{a_{n}s^{\alpha_{n}}+a_{n-1}s^{\alpha_{n-1}}+\ldots +a_{0}s^{\alpha_{0}}}$$

This study employed approximation using curve fitting and identification techniques, utilizing the Oustaloup method, an approach based on approximating functions of the following form:

(4) $$H(s)=s^{\mu },\;\;\mu \in R^{+}$$

The standard formulation of the Oustaloup filter is expressed as follows:

(5) H^(s)=G∏k=−NN1+sωk1+sωk′

The synthesis formula is given as follows:

(6) $$\begin{array}{c} \omega_{0}^{\prime }=\alpha^{-0.5}\omega_{u};\;\omega_{0}=\alpha^{0.5}\omega_{u};\;\frac{\omega_{k+1}^{\prime }}{\omega_{k}^{\prime }}=\frac{\omega_{k+1}}{\omega_{k}}=\alpha \eta >1;\\ \frac{\omega_{k+1}^{\prime }}{\omega_{k}^{\prime }}=\eta >0;\;\frac{\omega_{k}}{\omega_{k}^{\prime }}=\alpha >0;\;N=\frac{\log(\omega_{N}/\omega_{0})}{\log(\alpha \eta )};\;\mu =\frac{\log\alpha }{\log(\alpha \eta )} \end{array}$$

In Equations (4)–(6), *s* represents the complex frequency variable in the Laplace domain, *μ* represents the fractional order (*μ* ∈ R^+^), *G* represents the normalization gain factor, and *N* represents the approximation order. The approximation order *N* of the Oustaloup filter is a key parameter for balancing the system accuracy and implementation complexity. *N* determines the accuracy of the external circuit used to approximate fractional-order capacitors/inductors: a larger *N* enhances the frequency-domain approximation effect, while a smaller *N* simplifies the circuit. The recursive pole/zero frequencies are represented by ωₖ (poles) and ωₖ’ (zeros), with ω_0_ = α^0^·^5^ωᵢ’ and ω_0_’ = α^−0^·^5^ωᵢ’ establishing the central references. The geometric progression ratios are characterized by α (pole/zero spacing ratio) and η (bandwidth scaling factor), where αη > 1 ensures a proper frequency distribution. The unit gain frequency ωᵤ = $\sqrt{\omega ₕ\omega ₗ}$ is geometrically determined by ωₕ (high transition frequency) and ωₗ (low transition frequency), while ωₖ denotes the center frequencies of the operational bands. The fractional order μ is maintained through the logarithmic relationship μ = logα/log(αη).

Therefore, this study achieved integer-order biquadratic transfer function approximation for individual fractional-order electrical components using the Oustaloup filter algorithm.

Specifically, the fractional-order capacitance was approximated as follows:

(7) $$C_{\alpha_{2}}s^{\alpha_{2}}=0.08s^{0.82}\approx \frac{23.07s^{2}+43.00s+0.08}{s^{2}+537.54s+288.37}$$

The fractional-order inductance approximation was implemented as follows:

(8) $$\frac{1}{L_{\beta_{2}}s^{\beta_{2}}}=\frac{1}{0.28s^{0.88}}\approx \frac{0.0082s^{2}+5.43s+3.57}{s^{2}+1.52s+0.0023}$$

**Figure 13:**
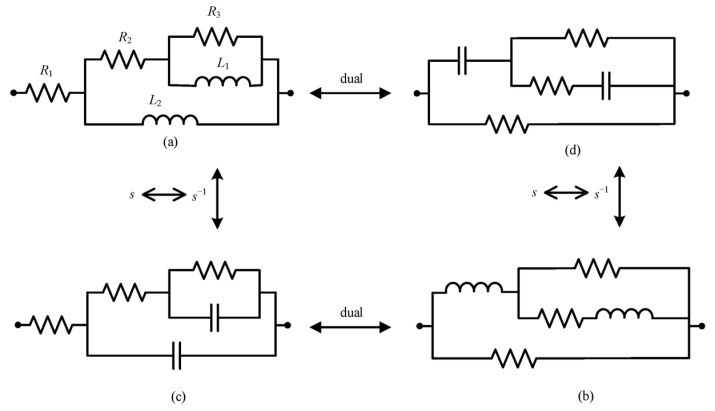
(**a**–**d**) The first step in the biquadratic admittance function network synthesis of five components.

**Figure 14:**
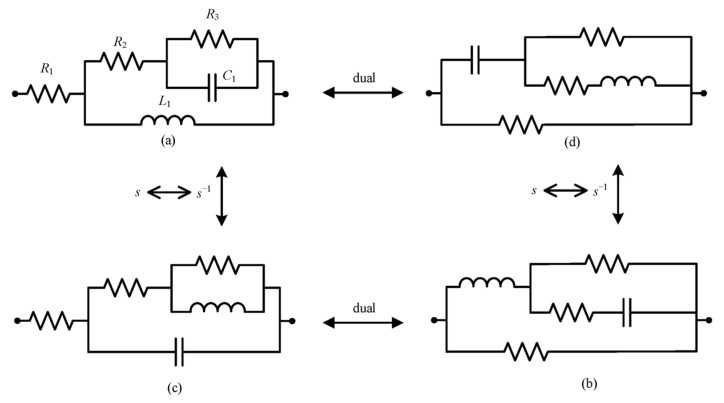
(**a**–**d**) The second step in the biquadratic admittance function network synthesis of five components.

The Bott–Duffin synthesis method [42], as a transformerless network realization approach, establishes a fundamental theorem in electrical network theory, which states that any positive real immittance function can be physically implemented with a finite set of resistors, capacitors, and inductors (see Figure 13 and Figure 14).

**Table 2:** Network synthesis realization conditions.

Network Topology	Necessary and Sufficient Conditions
Figure 13a,b	*K*_c_ ≤ 0, W ≥ 1
Figure 13c,d	*K*_c_ ≤ 0, W ≤ 1
Figure 14a	*K*_c_ ≥ 0, W ≥ 1, *λ*_c_ ≥ 0
Figure 14b	*K*_c_ ≥ 0, W ≥ 1, *λ*_c_ ≥ 0
Figure 14c	*K*_c_ ≥ 0, W ≤ 1, *λ*_c_ ≥ 0
Figure 14d	*K*_c_ ≥ 0, W ≤ 1, *λ*_c_ ≥ 0

**Figure 15:**
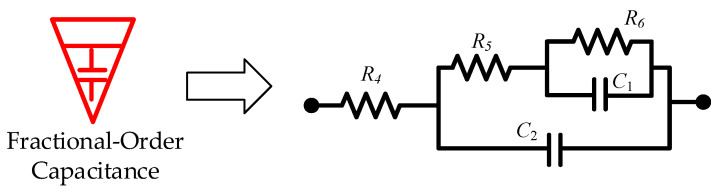
Fractional-order capacitance approximation circuit.

**Figure 16:**
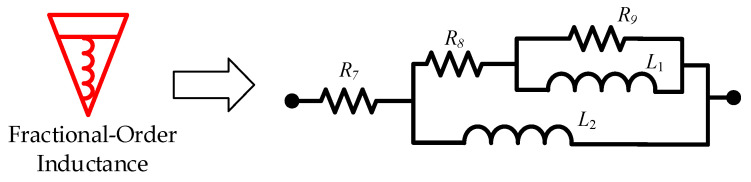
Fractional-order inductance approximation circuit.

By substituting the biquadratic admittance functions from Equations (7) and (8) into the realization conditions specified in Table 2, the electrical network structure shown below was derived. The corresponding component parameters for the fractional-order capacitance and inductor approximation circuits are detailed in Table 3 and Table 4, and Figure 15 and Figure 16, respectively. 

### 4.2. Simulation Verification of Integer-Order Approximation Circuits

By comparing the Bode plots of the ideal fractional-order circuit with those of the integer-order approximation circuit, we evaluated the consistency in both their magnitude-frequency and phase-frequency responses, thereby verifying the effectiveness of the designed approximation approach.

**Figure 17:**
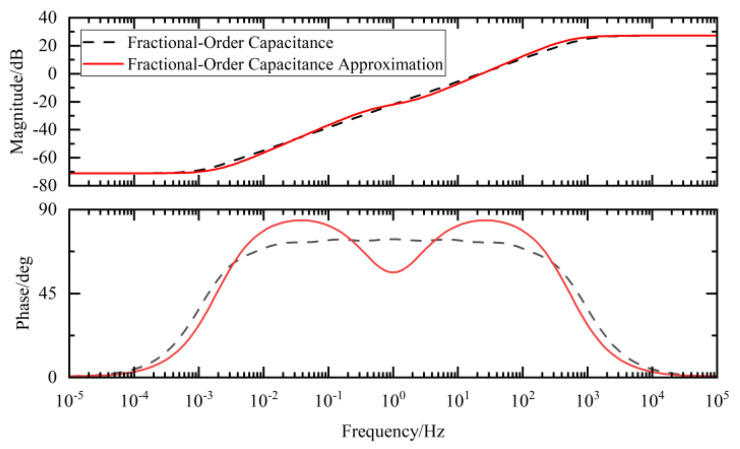
Fractional-order capacitance and its approximate Bode diagram.

**Figure 18:**
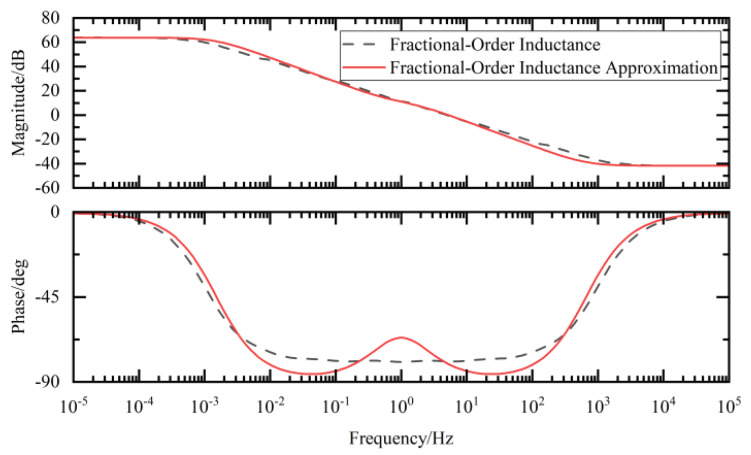
Fractional-order inductance and its approximate Bode diagram.

**Figure 19:**
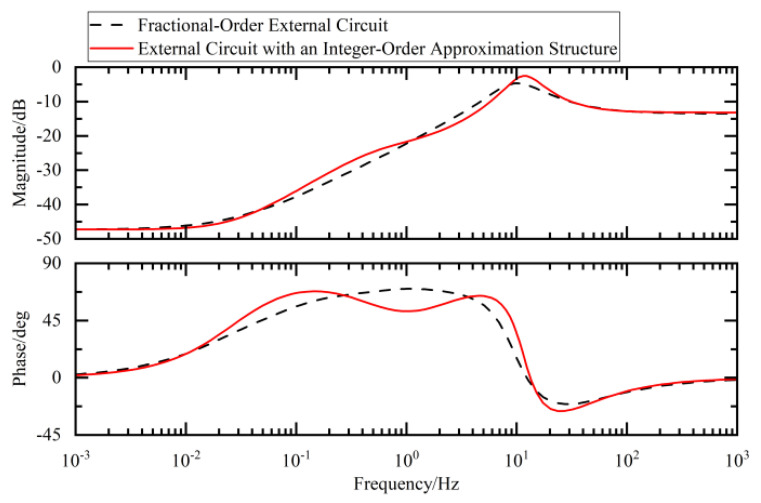
Fractional-order external circuit and its approximate Bode diagram.

In the Bode plots of the fractional-order external circuit shown in Figure 17, Figure 18 and Figure 19 in this paper, the results of the comparison between the values obtained using the integer-order approximation method and the theoretical values indicate that although there were slight differences in the frequency band of 10^−2^–10^2^ Hz, the overall variation trend was highly consistent. In-depth analysis revealed that within this frequency band, the magnitude approximation errors of the fractional-order capacitor and inductor were extremely small, while the phase deviations were mainly concentrated around 1 Hz. The magnitude and phase characteristics of the external circuit with an integer-order approximation structure were basically consistent with those of the ideal fractional-order system.

### 4.3. Comparison of Integer-Order Approximation Suspension’s Dynamic Performance Errors

#### 4.3.1. Comparison of Suspension’s Performance Regarding Time-Domain Errors

**Figure 20:**
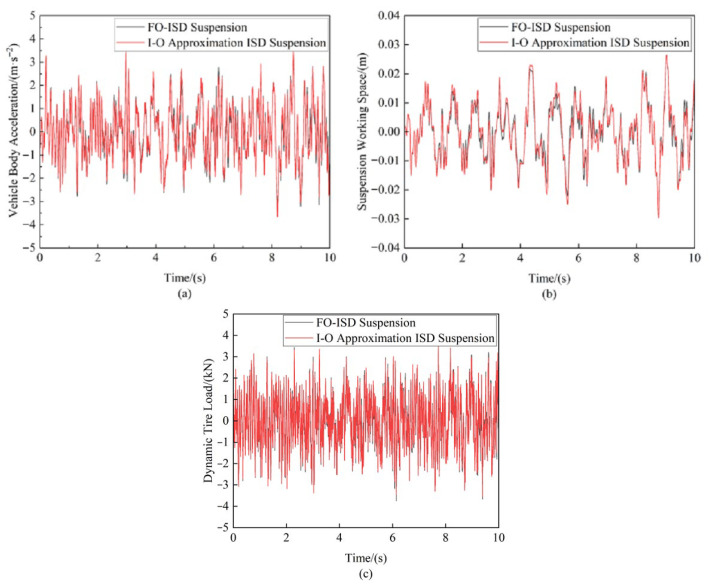
Suspension performance error comparison under random road input: (**a**) Vehicle body acceleration (**b**) Suspension working space (**c**) Dynamic tire load.

Figure 20 displays the suspension’s dynamic performance metrics when the vehicle traversed Class C road surfaces at 20 m/s, while Table 5 provides a comparative analysis of the RMS values for these metrics, specifically quantifying the approximation errors of the integer-order (I-O) approximation ISD suspension relative to those of its corresponding fractional-order (FO-ISD) suspension. The time-domain results in Figure 20 reveal an exceptional approximation fidelity for both the vehicle body acceleration and dynamic tire load, with RMS errors below 1%, as evidenced in Table 6. In contrast, the suspension working space showed the highest approximation error, with an RMS value of 7.69%. Nevertheless, it still achieved a substantial improvement over conventional passive suspension systems. The performance data for passive suspensions used in this comparison were derived from reference [19].

#### 4.3.2. Frequency-Domain Error Comparison of Suspension Performance

**Figure 21:**
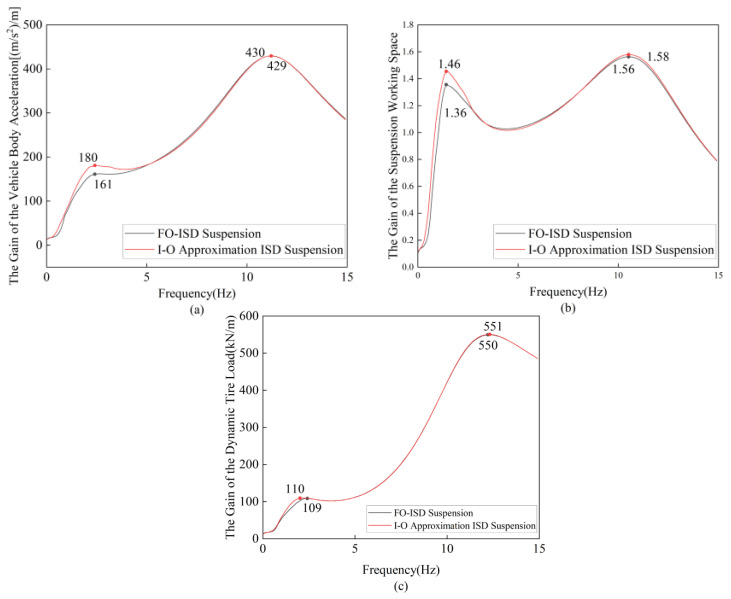
Comparison of suspension gain errors in frequency domain: (**a**) The gain of the vehicle body acceleration (**b**) The gain of the suspension working space (**c**) The gain of the dynamic tire load.

Figure 21 presents a comparison of the dynamic performance indicators of the FO-ISD suspension and its corresponding I-O approximation ISD suspension under random road excitation in the frequency domain. Table 6 provides a comparison of the low-frequency (LF) and high-frequency (HF) peak errors in the suspension performance metrics. The results indicate that the integer-order approximation exhibited suboptimal accuracy in the LF range, particularly for the vehicle body acceleration and suspension working space. Specifically, the LF peak errors for these metrics were 11.80% and 7.35%, respectively. However, compared to the traditional passive suspension, the I-O approximation ISD suspension still achieved significant improvements, reducing the LF peaks of the vehicle body acceleration and suspension working space by 11.76% and 38.14%, respectively. Outside the 1–4 Hz range, the I-O approximation ISD suspension demonstrated better approximation accuracy, with HF peak errors of below 1%. Additionally, the dynamic tire load exhibited highly satisfactory approximation performance across the entire 0–15 Hz frequency band.

#### 4.3.3. Impulse Road Excitation Error Comparison of Suspension Performance

**Figure 22:**
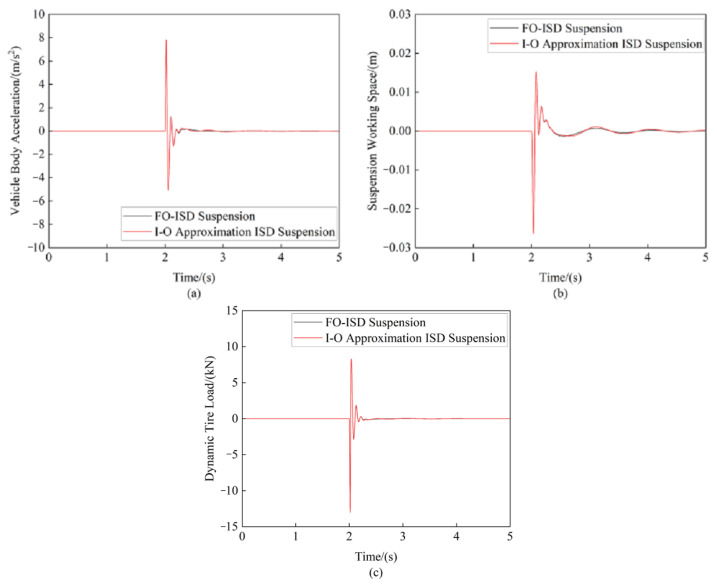
Comparison of suspension indices under pulsed road input: (**a**) Vehicle body acceleration (**b**) Suspension working space (**c**) Dynamic tire load.

Figure 22 presents a comparative analysis of the suspension’s dynamic performance indices when the vehicle traversed a speed bump with a 0.05 m height and 0.3 m width at 10 m/s. Table 7 provides detailed comparisons of the peak-to-peak (PTP) errors for each performance metric.

## 5. Experimental Verification

As established in Section 4, the Oustaloup filter algorithm was successfully employed to approximate the fractional-order electrical components, ultimately yielding an optimal I-O approximation ISD suspension. This section describes how we conducted bench testing by replacing the FO-ISD suspension with its corresponding I-O approximation ISD suspension for experimental validation.

### 5.1. Experimental Device

**Figure 23:**
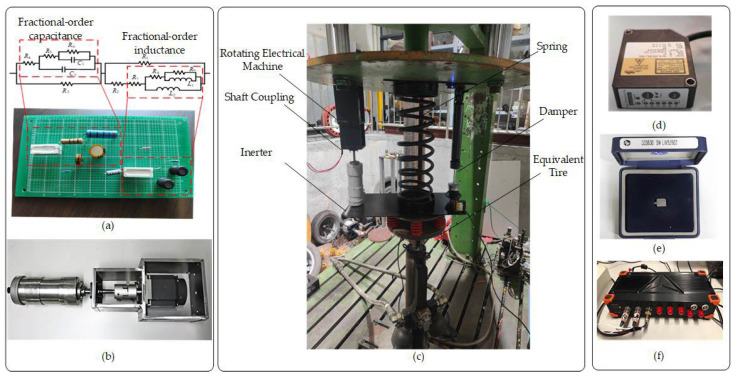
(**a**–**f**) Experimental verification of automotive mechatronic ISD suspension.

This study presents the experimental validation of an automotive mechatronic ISD suspension incorporating a fractional-order biquadratic transfer function. The test platform configuration is illustrated in Figure 23. Figure 23a shows the integer-order equivalent circuit implemented for fractional-order elements using Oustaloup filter approximation. A physical prototype of the ball-screw mechatronic inerter device is displayed in Figure 23b. The suspension test rig was constructed using an INSTRON 8800 digital hydraulic servo vibration excitation system, as depicted in Figure 23c. The measurement system comprised three specialized instruments for critical parameter acquisition. The data acquisition unit shown in Figure 23f was the core of the real-time signal acquisition system. It used an NI cDAQ-9188 system, featuring a 1 kHz sampling rate and 24-bit resolution, which could achieve strict inter-channel synchronization and support eight-channel synchronous sampling, fully meeting the requirements for the acquisition of random road excitation signals. Specifically, the vehicle body acceleration signals output by the accelerometers shown in Figure 23d and the suspension working space data collected by the laser displacement sensors shown in Figure 23e were both transmitted to this acquisition unit for processing, while the dynamic tire load information was synchronously obtained at the control end of the vibration excitation table.

### 5.2. Random Road Excitation Testing

**Figure 24:**
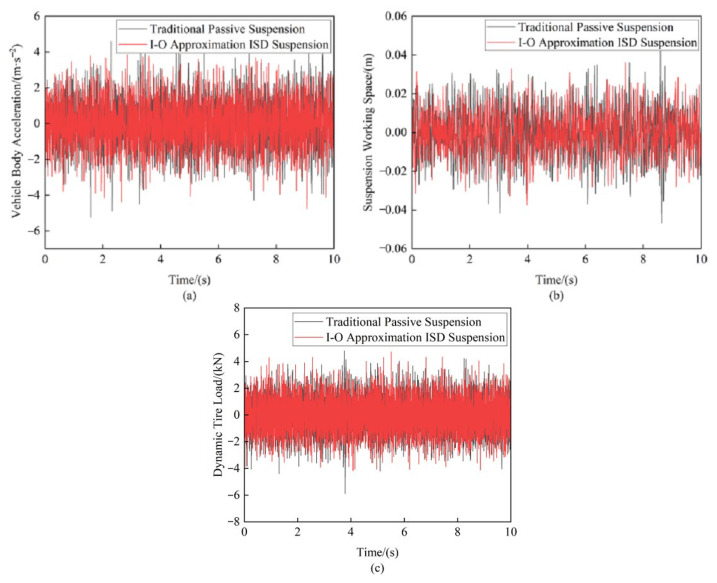
Comparison of suspension performance indicators at 20 m/s: (**a**) Vehicle body acceleration (**b**) Suspension working space (**c**) Dynamic tire load.

The experimental study applied Class C random road profile excitation to the vibration test platform to comparatively assess the vertical vibration responses of both the I-O Approximation ISD suspension and the traditional passive suspension system under three representative vehicle speeds: 10 m/s, 20 m/s, and 30 m/s (see Figure 24 and Table 8).

The experimental results effectively validated the performance advantages of the I-O approximation ISD suspension. At a vehicle speed of 20 m/s, the I-O approximation ISD suspension achieved significant improvements in all key metrics compared to the traditional passive suspension. The RMS value of the vehicle body acceleration showed a 7.86% reduction. The suspension working space improved remarkably, with a 17.45% decrease in its RMS values. The dynamic tire load exhibited a 2.26% reduction in its RMS measurements. These quantitative results validate the superior vibration isolation performance of the fractional-order design. Moreover, the experimental results are in excellent agreement with the FO-ISD simulation data reported in reference [19], thereby further confirming the performance advantages of the fractional-order biquadratic transfer function-based mechatronic ISD suspension.

## 6. Discussion

This paper presented an implementation of a vehicle mechatronic ISD suspension system based on a fractional-order biquadratic network. The Oustaloup filter algorithm was employed to approximate a fractional-order capacitor and fractional-order inductor using integer-order biquadratic transfer functions, and the specific approximation results obtained through five-element passive network synthesis. Simulation analyses were conducted to compare the FO-ISD suspension with its corresponding I-O approximation ISD suspension, verifying the effectiveness of the approximated integer-order suspension structure. Finally, bench tests confirmed the performance advantages of the I-O approximation ISD suspension. The proposed fractional-order biquadratic transfer function-based mechatronic ISD suspension showed superior performance while offering innovative theoretical and practical solutions for advanced suspension design.

Future research will focus on exploring the application of higher-order fractional orders in suspensions to expand the frequency-domain vibration isolation range, integrating intelligent control algorithms for the real-time parameter adjustment of fractional-order suspensions, and conducting experimental verification under complex operating conditions.

## Figures and Tables

**Table 1:** Suspension and external circuit parameters.

Parameters	Values
Sprung mass, *m_s_*	675/kg
Unsprung mass, *m_u_*	62.5/kg
Tire stiffness, *k_t_*	290,000/N∙m^−1^
Spring stiffness, *k*	53,100/N∙m^−1^
Damper coefficient, *c*	1000/N∙s∙m^−1^
Inertance, *b*	10/kg
*A*	0.21
*B*	3.70
*C*	0.01
*D*	0.20
*F*	0.52
*G*	2.88
*H*	49.13
Resistance, *R*_1_	4.69/Ω
Resistance, *R*_2_	0.15/Ω
Resistance, *R*_3_	246.86/Ω
Fractional-order capacitance, $C_{\alpha_{2}}$	0.08/F
Fractional order of capacitance, α_2_	0.82
Fractional-order inductance, $L_{\beta_{2}}$	0.28/H
Fractional order of inductance, *β*_2_	0.88

**Table 3:** Circuit element parameters for fractional-order capacitance approximation.

Parameters	Values
Resistance *R*_4_	0.00028/Ω
Resistance *R*_5_	17.55/Ω
Resistance *R*_6_	3587.08/Ω
Capacitance *C*_1_	0.107/F
Capacitance *C*_2_	0.043/F

**Table 4:** Circuit element parameters for fractional-order inductance approximation.

Parameters	Values
Resistance *R*_7_	0.00064/Ω
Resistance *R*_8_	0.50/Ω
Resistance *R*_9_	121.73/Ω
Inductance *L*_1_	0.33/H
Inductance *L*_2_	0.42/H

**Table 5:** Suspension performance error comparison.

	FO-ISD Suspension	I-O Approximation ISD Suspension	Error
RMS of Vehicle Body Acceleration/(m·s^−2^)	1.1632	1.1745	0.97%
RMS of Suspension Working Space/(m)	0.0091	0.0098	7.69%
RMS of Dynamic Tire Load/(N)	1155.9	1160.7	0.42%

**Table 6:** Suspension frequency-domain peak error comparison.

		FO-ISD Suspension	I-O Approximation ISD Suspension	Error
The Gain of the Vehicle Body Acceleration/[(m·s^−2^)/m]	LF	161	180	11.80%
	HF	429	430	0.23%
The Gain of the Suspension Working Space	LF	1.36	1.46	7.35%
	HF	1.56	1.58	1.28%
The Gain of the Dynamic Tire Load/(kN/m)	LF	109	110	0.92%
	HF	550	551	0.18%

**Table 7:** Comparison of peak-to-peak errors in suspension’s performance indices.

	FO-ISD Suspension	I-O Approximation ISD Suspension	Error
PTP value of the vehicle body acceleration/(m·s^−2^)	12.8691	12.8370	−0.25%
PTP value of the suspension working space/(m)	0.0416	0.0417	0.24%
PTP value of the dynamic tire load/(kN)	21.314	21.304	−0.05%

**Table 8:** Data from suspension test under random road input.

Suspension	Velocity *u*/(m/s)	RMS of Vehicle Body Acceleration/(m·s^−2^)	RMS of Suspension Working Space/(m)	RMS of Dynamic Tire Load/(N)
Traditional Passive Suspension (Experiment)	10	0.9062	0.0081	844.0
	20	1.2641	0.0112	1184.5
	30	1.5178	0.0133	1435.1
I-O Approximation ISD Suspension (Experiment)	10	0.9500	0.0075	913.8
	20	1.2900	0.0102	1301.9
	30	1.5700	0.0121	1576.4
Optimization (Experiment)	10	8.65%	17.26%	2.60%
	20	7.86%	17.45%	2.26%
	30	7.10%	17.48%	1.93%

## Data Availability

All the pertinent data are available in this article.

## Abbreviations

The following abbreviations are used in this manuscript:

ISD	Inerter–spring–damper
RMS	Root mean square
LF	Low-frequency
HF	High-frequency
PTP	Peak-to-peak
I-O	Integer-order
FO	Fractional-order
